# Genotype-by-environment interaction in Holstein heifer fertility traits using single-step genomic reaction norm models

**DOI:** 10.1186/s12864-021-07496-3

**Published:** 2021-03-17

**Authors:** Rui Shi, Luiz Fernando Brito, Aoxing Liu, Hanpeng Luo, Ziwei Chen, Lin Liu, Gang Guo, Herman Mulder, Bart Ducro, Aart van der Linden, Yachun Wang

**Affiliations:** 1 Key Laboratory of Animal Genetics, Breeding and Reproduction, MARA, National Engineering Laboratory of Animal Breeding, College of Animal Science and Technology, China Agricultural University, Beijing, 100193 China; 2 Animal Breeding and Genomics Group, Wageningen University & Research, P.O. Box 338, Wageningen, AH 6700 the Netherlands; 3 Animal Production System Group, Wageningen University & Research, P.O. Box 338, Wageningen, AH 6700 the Netherlands; 4 Department of Animal Sciences, Purdue University, West Lafayette, Indiana 47907 USA; 5 Center for Quantitative Genetics and Genomics, Aarhus University, 8830 Tjele, Denmark; 6 Beijing Dairy Cattle Center, Beijing, 100192 China; 7 Beijing Sunlon Livestock Development Co. Ltd, Beijing, 100176 China; 8 Cooperation CRV, Arnhem, AL 6800 the Netherlands

**Keywords:** Heifer, Heat stress, Genotype-by-environment interaction, Reaction norm, Single-step GWAS

## Abstract

**Background:**

The effect of heat stress on livestock production is a worldwide issue. Animal performance is influenced by exposure to harsh environmental conditions potentially causing genotype-by-environment interactions (G × E), especially in highproducing animals. In this context, the main objectives of this study were to (1) detect the time periods in which heifer fertility traits are more sensitive to the exposure to high environmental temperature and/or humidity, (2) investigate G × E due to heat stress in heifer fertility traits, and, (3) identify genomic regions associated with heifer fertility and heat tolerance in Holstein cattle.

**Results:**

Phenotypic records for three heifer fertility traits (i.e., age at first calving, interval from first to last service, and conception rate at the first service) were collected, from 2005 to 2018, for 56,998 Holstein heifers raised in 15 herds in the Beijing area (China). By integrating environmental data, including hourly air temperature and relative humidity, the critical periods in which the heifers are more sensitive to heat stress were located in more than 30 days before the first service for age at first calving and interval from first to last service, or 10 days before and less than 60 days after the first service for conception rate. Using reaction norm models, significant G × E was detected for all three traits regarding both environmental gradients, proportion of days exceeding heat threshold, and minimum temperature-humidity index. Through single-step genome-wide association studies, *PLAG1*, *AMHR2*, *SP1*, *KRT8*, *KRT18*, *MLH1*, and *EOMES* were suggested as candidate genes for heifer fertility. The genes *HCRTR1*, *AGRP*, *PC*, and *GUCY1B1* are strong candidates for association with heat tolerance.

**Conclusions:**

The critical periods in which the reproductive performance of heifers is more sensitive to heat stress are trait-dependent. Thus, detailed analysis should be conducted to determine this particular period for other fertility traits. The considerable magnitude of G × E and sire re-ranking indicates the necessity to consider G × E in dairy cattle breeding schemes. This will enable selection of more heat-tolerant animals with high reproductive efficiency under harsh climatic conditions. Lastly, the candidate genes identified to be linked with response to heat stress provide a better understanding of the underlying biological mechanisms of heat tolerance in dairy cattle.

**Supplementary Information:**

The online version contains supplementary material available at 10.1186/s12864-021-07496-3.

## Background

In modern dairy cattle farms, female fertility is of great importance, due to its close relationship with reproductive management, veterinary treatments, involuntary culling and, consequently, the farm profitability [1]. However, as widely emphasized in previous studies [2–4], the low heritability estimates for fertility traits and unfavorable genetic correlations with milk production traits have led to reduced genetic progress in female fertility. Moreover, the increase of joint genetic evaluation (and breeding) across farms located in various geographical regions emphasizes the role that genotype-by-environment interactions (G × E) [5] might play, and consequently, selection of animals (especially bulls) that have progeny with high performance even in challenging environments. Significant G × E for female fertility traits have been detected in several Holstein populations around the world, where the “E” were the production system and grass ratio of feed [6], and herd reproduction level [7]. However, the investigation of other important environmental indicators such as climatic variables remain scarce.

With global warming and climatic change, heat stress has become an issue for livestock production in many countries around the world [8]. The temperature and humidity index (THI) is often used as an environmental indicator to assess heat stress conditions in dairy cattle [9]. It is widely accepted that dairy cows start to experience mild heat stress when THI surpasses 72 [10]. Studies of the North American Holstein population have shown that heat conditions can lead to 165 kg loss of milk yield annually and 0.4% reduction in milk fat percentage [11, 12], 0.85 kg decrease in feed intake with one unit increase in air temperature [13], and about 15% decrease in conception rate when THI surpasses 72 [14].

The average daily THI in many regions of the world exceed 72 throughout most summer period days, indicating that dairy cattle located in these regions may suffer from mild to severe heat stress [15]. For instance, in Beijing (China), THI fluctuates substantially within a day, that is, extremely high THI in the afternoon and dramatically falls to a thermoneutral level in the evening. The difference in hourly THI within a day can be up to 30 THI units during the late summer, but the daily average is usually only relatively “mild” (Suppl. File 1). In this case, simply using the daily average of THI may lead to the underestimation of the impact of heat stress. In addition to the timing of the day, for dairy cows, the time of its reproductive period may also influence the response to heat stress. Fertility performance may be compromised when an animal experiences heat stress in certain physiological stages. Several studies have demonstrated that the conception rate of dairy cows decreased when they experienced heat stress before and after insemination [16–18], which highlights the role of the critical period of exposure to heat conditions. To the best of our knowledge, no studies have identified the most influential (critical) period for fertility traits due to their complex characteristics. However, this is of utmost value for incorporating G × E models in genetic and genomic evaluations for improved fertility.

Reaction norm models (RNM) are widely used to detect G × E when the differences in environments can be measured by a continuous environmental gradient (EG) [5]. In RNM, the breeding value of an animal is partitioned into an environment-independent part (intercept) and an environment-dependent part (slope). The relationship matrix of the RNM can be structured either by pedigree and using the pedigree-based Best Linear Unbiased Prediction (BLUP), or by combining both pedigree and genomic information and using the single-step genomic BLUP (ssGBLUP) method [19, 20]. On the basis of ssGBLUP, Wang et al. [21] proposed a method termed single-step GWAS (ssGWAS) to obtain genomic marker effects from genomic estimated breeding values (GEBV). Markers related to the intercept and slope of the reaction norms can be mapped by applying ssGWAS procedures.

The main objectives of this study were to: (1) explore the most heat-sensitive periods for three heifer fertility traits: age at first calving – AFC, interval from first to last service – IFL, and conception rate of first service – CR; (2) detect G × E for heifer fertility traits using RNM with pedigree-genomic combined relationship matrix; and (3) unravel genomic regions contributing to heat tolerance and heifer fertility traits in high-producing Holstein cattle.

## Results

### Descriptive statistics

The summary statistics for heifer fertility traits are shown in Table 1. Large phenotypic variation was detected, especially for IFL (coefficient of variation equals to 1.89) and CR (coefficient of variation equals to 0.83). The genetic parameters estimated using the conventional animal model, which were relatively low, are also provided in Table 1.

**Table 1:** Descriptive statistics of heifer fertility traits and genetic parameters estimated using pedigree-based animal models

Trait^a^	N	Mean	SD	CV	Min	Max	${\upsigma}_{\mathrm{a}}^2$(SE)	${\upsigma}_e^2$(SE)	h^2^(SE)
AFC (days)	56,998	769.05	74.06	0.10	505	1100	794.40 (54.27)	4035.00 (46.35)	0.16 (0.011)
IFL (days)	56,998	29.25	55.17	1.89	0	365	190.42 (22.33)	2740.10 (23.82)	0.06 (0.007)
CR (0 or 1 scale)	56,998	0.59	0.49	0.83	0	1	6.61e-3 (1.11e-3)	2.16e-1 (1.57e-3)	0.03 (0.005)

^a^ *AFC* Age at first calving, *IFL* Interval from first to last service, *CR* Conception rate of first service

### Critical period selection for each environmental gradient scenario

Two heat related EGs were used in the current study: 1) the number of days that exceeded the THI threshold in the evaluated critical period (prop-EG); 2) the minimum THI for each day of the candidate period (mTHI-EG). To avoid the underestimation of the heat stress effect, the days in which the hourly THI was higher than 72 for six continuous hours were considered as heat-stress days for prop-EG. The Akaike Information Criterion (AIC) [22] was obtained for various time combinations to select the best fit period for each trait.

**Fig. 1:**
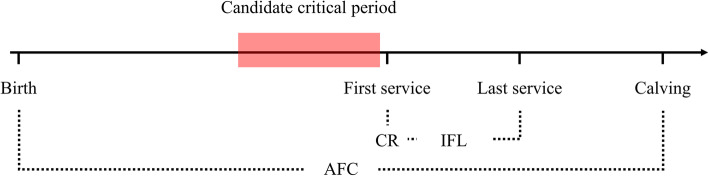
Reproductive events and the definition of critical period in heifers. The red rectangle represents the critical period, defined as the time period for which heifers are likely to suffer from heat stress. AFC = age at first calving, IFL = interval from first to last service, CR = conception rate of first service

The critical periods (Fig. 1) selected for each trait and EG under scenario one (S1) and scenario two (S2) are listed in Table 2. The same 60 days, from 30 days before the first insemination to 30 days after the first insemination, were chosen as the control period for S1. For S2, critical periods ranged from 30 to 70 days, of which only the period (− 90, − 30) for IFL was the same for both EGs. Only the critical periods of CR end after the first service (60 or 30 days). The detailed results of the AIC values for the 19 tested combinations are presented in Supp. File 2.

**Table 2:** The critical periods selected for each fertility trait and environmental gradient (EG) scenario

EG^a^	Trait^b^	Scenario^c^	Number of days	Period^d^
Prop-EG	AFC	S1	60	(−30, 30)
		S2	60	(−90, −30)
	IFL	S1	60	(−30, 30)
		S2	60	(−90, −30)
	CR	S1	60	(−30, 30)
		S2	70	(−10, 60)
mTHI-EG	AFC	S1	60	(−30, 30)
		S2	30	(−90, −60)
	IFL	S1	60	(−30, 30)
		S2	60	(−90, −30)
	CR	S1	60	(−30, 30)
		S2	40	(−10, 30)

^a^ *prop-EG* The number of days that exceeded the threshold temperature humidity index in the period, *mTHI-EG* Minimum temperature-humidity index for each day of the period

^b^ *AFC* Age at first calving, *IFL* Interval from the first to last service, *CR* Conception rate of first service

^c^ *S1* control critical period, *S2* periods selected based on the Akaike’s information criterion

^d^ Periods were counted based on the first service day; minus means before and plus means after

The definitions of two types of EGs had some overlaps. For example, prop-EG would be recorded as 1 if minimum THI of all the days in critical period were above 67.02 (Table 3). To calculate the overlap rate between prop-EG and mTHI-EG, top animals sorted by genomic estimated breeding values (gEBV), with estimation accuracy greater than 0.4 (average accuracy for the three traits), were chosen for each trait with regards to each EG. When using the ***H*** matrix (hybrid pedigree-genomic relationship matrix), approximately 75% of the heifers were the same in both scenarios for AFC and IFL, but relatively low (29.63% ~ 65.82%) overlap rates were observed in CR (Table 3). Similar results were found when using the (pedigree-based) ***A*** matrix (Supp. File 3).

**Table 3:** The proportion of overlapped top 1% heifers^a^ when using prop-EG and mTHL-EG as environmental gradients (EGs) in reaction norm models (RNM) with the ***H*** matrix in Holstein cattle

EG^b^		AFC^c^		IFL		CR	
Prop-EG	mTHI-EG	S1^d^	S2	S1	S2	S1	S2
0.2	43.03	76.47%	82.69%	72.92%	64.37%	29.63%	36.63%
0.4	48.29	84.57%	87.35%	81.24%	77.69%	59.60%	51.50%
0.6	52.34	88.24%	82.02%	85.68%	78.91%	65.82%	60.93%
0.8	55.54	83.02%	75.47%	80.69%	75.47%	65.70%	62.93%
1	67.02	82.24%	76.03%	79.69%	74.92%	64.04%	60.27%

^a^ Heifers were selected based on gEBV and accuracy of estimation (> 0.4)

^b^ *prop-EG* using the number of days that exceeding the threshold temperature humidity index in the period as EG, *mTHI-EG* using the minimum temperature humidity index of a day of the critical period as EG

^c^ *AFC* Age at first calving, *IFL* Interval from the first to last service, *CR* Conception rate of first service

^d^ *S1* reference period, *S2* periods selected based on the Akaike’s information criterion

### (co) variance components and G × E

The estimates of (co) variance components obtained from RNMs with different kinship matrices (***A*** or ***H***) were similar for all traits analyzed. The correlation coefficients between the intercept and slope for each trait were all negative and ranged from − 0.25 (IFL in S1 of prop-EG) to − 0.98 (CR in both S1 and S2 of mTHI-EG) when using the ***H*** matrix (Table 4). Furthermore, the absolute value of coefficients estimated using prop-EG were relatively smaller than those using mTHI-EG, especially for AFC and IFL. The genetic parameters estimated based on the ***A*** matrix are shown in Supp. File 4.

**Table 4:** Variances of the intercept (${\upsigma}_{a_0}^2$) and slope (${\upsigma}_{a_1}^2$), the covariance between the intercept and slope (${\sigma}_{a_0{a}_1}$), residual variance (${\upsigma}_e^2$), and genetic correlation between the intercept and slope (${r}_{a_0{a}_1}$), with their standard errors in parentheses, estimated using reaction norm models with ***H*** matrix in Holstein cattle

EG^a^	Trait^b^	Scenario^c^	${\upsigma}_{a_0}^2$	${\upsigma}_{a_1}^2$	${\sigma}_{a_0{a}_1}$	${\upsigma}_e^2$	${r}_{a_0{a}_1}$
Prop-EG	AFC	S1	971.97 (62.74)	0.53 (0.05)	−9.62 (1.41)	3413.70 (45.80)	−0.43 (0.02)
		S2	963.72 (60.99)	0.96 (0.06)	−14.05 (1.62)	3203.00 (44.60)	−0.46 (0.01)
	IFL	S1	218.16 (26.82)	0.25 (0.03)	−1.80 (0.68)	2456.90 (25.14)	−0.25 (0.03)
		S2	236.35 (27.59)	0.51 (0.03)	−4.98 (0.87)	2323.10 (24.76)	−0.46 (0.02)
	CR	S1	1.01e-2 (1.60e−3)	1.60e-5 (2.00e-6)	-3.35e-4 (5.20e-5)	1.99e-1 (1.61e-03)	−0.83 (0.05)
		S2	1.12e-2 (1.71e−3)	1.30e-5 (2.00e-6)	-3.23e-4 (4.70e-5)	1.98e-1 (1.62e-3)	−0.84 (0.05)
mTHI-EG	AFC	S1	2735.10 (233.57)	1.37 (0.12)	−51.60 (5.10)	3396.80 (45.06)	−0.84 (0.03)
		S2	3950.90 (271.77)	2.06 (0.12)	− 81.46 (5.69)	3194.70 (43.73)	−0.90 (0.03)
	IFL	S1	1073.60 (126.34)	0.78 (0.07)	−26.15 (2.92)	2417.70 (25.10)	−0.90 (0.04)
		S2	1591.40 (143.27)	1.24 (0.08)	−41.85 (3.35)	2309.10 (24.37)	−0.94 (0.03)
	CR	S1	7.78e-2 (9.60e-3)	4.30e-5 (5.00e-6)	−1.81e-3 (2.14e-4)	1.99e-1 (1.58e-3)	−0.98 (0.06)
		S2	8.90e-2 (1.03e-2)	4.50e-5 (5.00e-6)	−1.96e-3 (2.19e-4)	1.98e-1 (1.59e-3)	−0.98 (0.05)

^a^ *prop-EG* the number of days that exceeding the threshold temperature humidity index in the period, *mTHI-EG* Minimum temperature humidity index for each day of the period

^b^ *AFC* Age at first calving, *IFL* Interval from the first to last service, *CR* Conception rate of first service

^c^ *S1* reference period, *S2* periods selected based on the Akaike’s information criterion

**Fig. 2:**
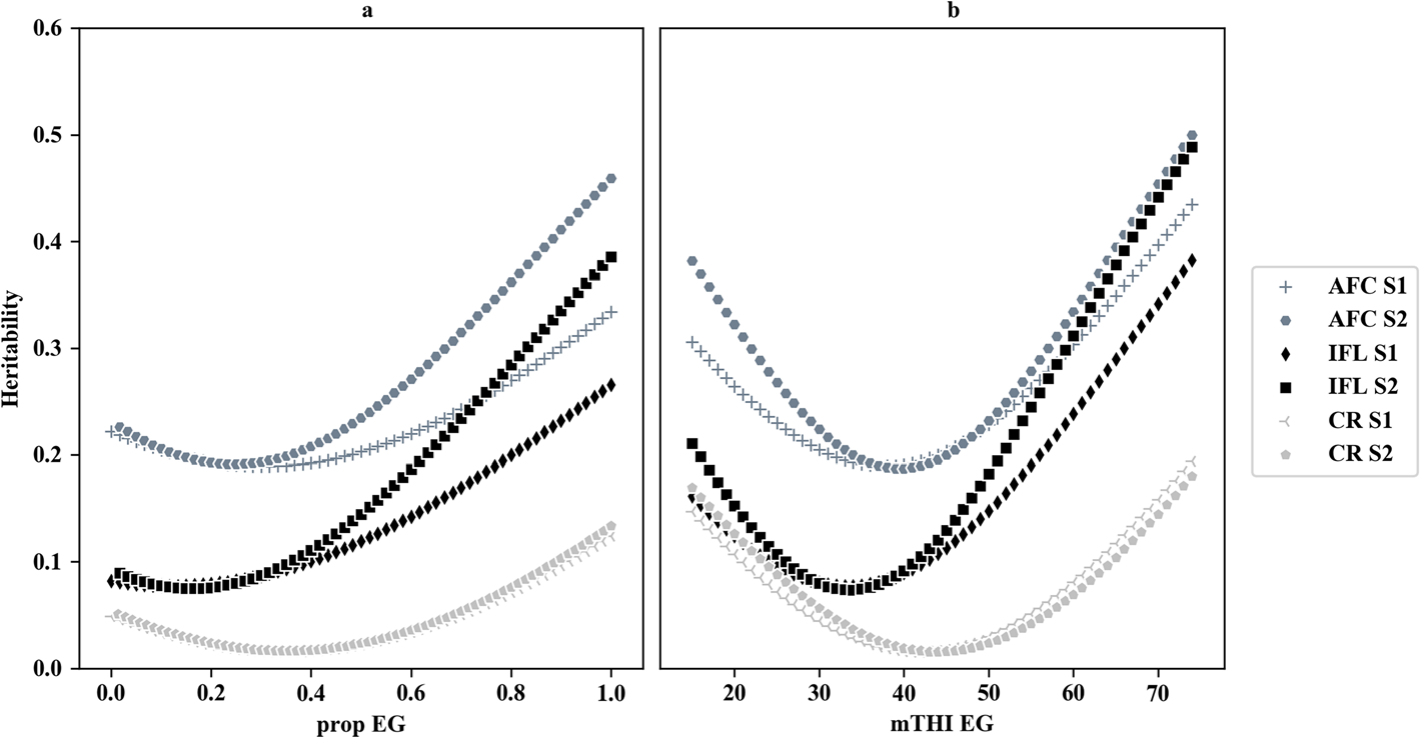
Heritabilities estimated based on reaction norm models with the matrix ***H*** for different traits using **a** prop-EG or **b** mTHI-EG as environmental gradient. For **a**, the x-axis is the proportion of days exceeding the threshold with a range of 0 to 1; while for **b**, the x-axis is the minimum THI with a range of 15 to 75

Heritabilities estimated from genomic RNM using prop-EG and mTHI-EG are presented in Fig. 2. Generally, AFC had the highest heritability estimates, whereas CR was the least heritable across all EGs. The pattern of the heritability curves were similar when using different relationship matrices but differed across EGs. The curve patterns were quadratic for mTHI-EG, indicating that the highest heritabilities were generally observed in either cold (mTHI-EG < 20) or heat-stress environments (mTHI-EG > 72). However, the patterns were flatter when prop-EG was used, and the highest heritabilities only appeared in heat stress conditions. Similar curve patterns were observed when using the ***A*** matrix (Suppl File 5).

**Fig. 3:**
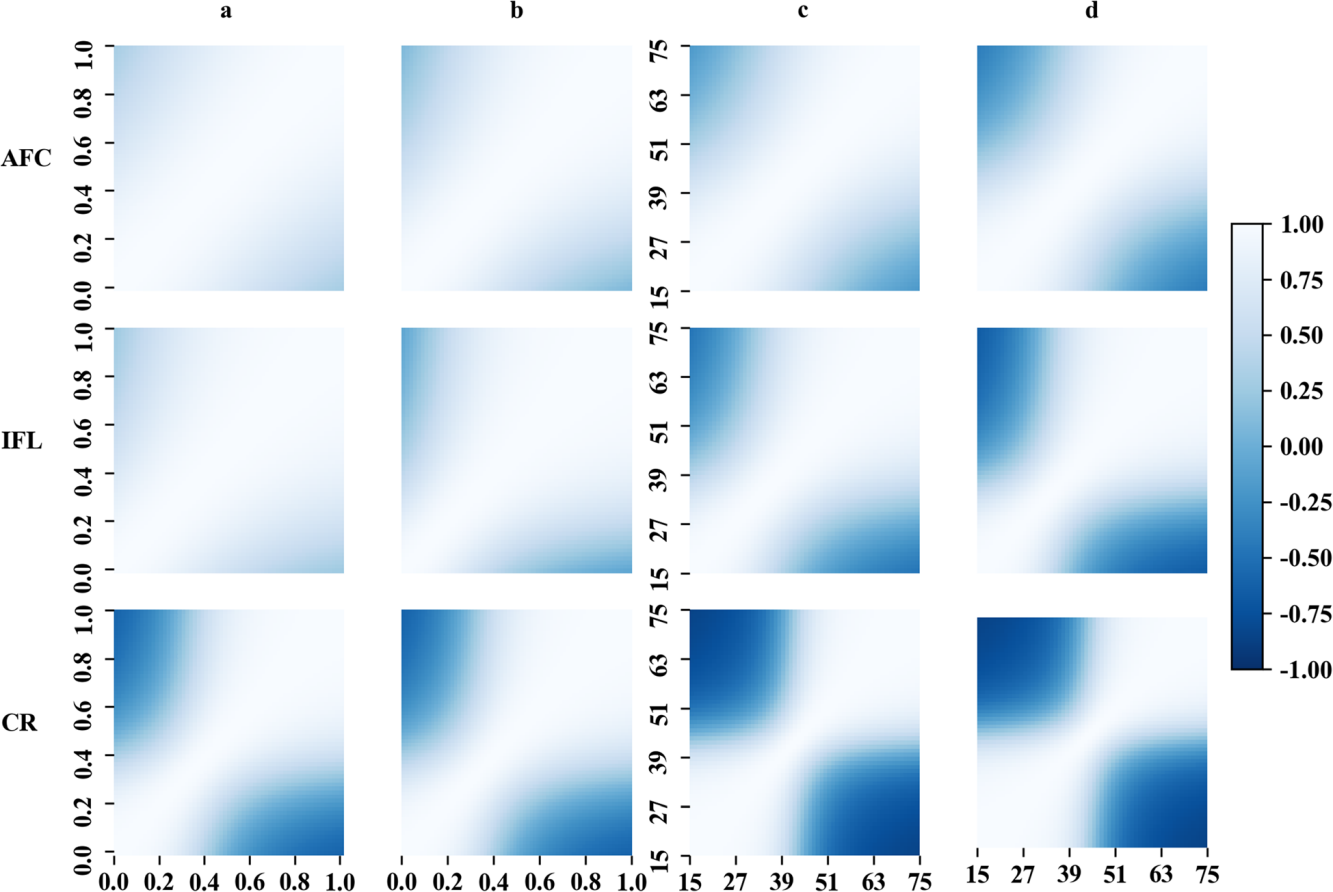
Genetic correlations estimated by reaction norm models (RNMs) with the matrix ***H***. The color indicates the magnitude of the genetic correlation. **a** Correlations between different levels of prop-EG estimated from RNM under S1. The x-axis and y-axis are the proportion of days exceeding the threshold, ranging from 0 to 1. **b** Correlations between different levels of prop-EG estimated from RNM under S2. The x-axis and y-axis are the proportion of days exceeding the threshold, ranging from 0 to 1. **c** Correlations between different levels of mTHI-EG estimated from RNM under S1. The x-axis and y-axis are the minimum THI, ranging from 15 to 75. **d** Correlations between different levels of mTHI-EG estimated from RNM under S2. The x-axis and y-axis are the minimum THI, ranging from 15 to 75

As shown in Table 4, the variance of the slope for all traits was significantly different from zero based on a one-tailed test (*P* < 0.01), indicating the existence of G × E. Genetic correlations between different EGs, from RNM with the ***H*** matrix, are shown in Fig. 3. In general, the more divergent EGs were less correlated. More negative coefficients of correlation were obtained for AFC and IFL when the mTHI-EG was used in comparison to prop-EG. This is consistent with much stronger correlation between the intercept and slope being observed when using mTHI-EG as EG compared to using prop-EG as EG. Similar patterns were also observed when fitting the ***A*** matrix (Suppl File 6).

Among the top sires with more than 20 daughters with phenotypes, the number of sires overlapping across the two EGs, reflecting the magnitude of the re-ranking of sires, are listed in Table 5. The number of common sires decreased as the EGs became more divergent, especially for CR (e.g., from 11 to 1 in S2 of prop-EG). The magnitude of re-ranking increased when using mTHI-EG (only 3 common sires across all environmental combinations).

**Table 5:** The number of common animals among the top 50 sires between 2 levels of environmental gradients (EGs)

EG^a^	Trait^b^	Scenario^c^	1 vs. 99%^d^	5 vs. 95%	10 vs. 90%	25 vs. 75%
Prop-EG	AFC	S1	18	20	21	28
		S2	13	14	17	28
	IFL	S1	18	20	21	29
		S2	12	14	17	27
	CR	S1	0	0	1	7
		S2	1	1	3	11
mTHI-EG	AFC	S1	5	8	15	25
		S2	3	4	9	24
	IFL	S1	3	5	6	22
		S2	0	0	2	22
	CR	S1	0	0	0	3
		S2	0	0	0	3

^a^ *prop-EG* the number of days that exceeding the threshold temperature humidity index in the period, *mTHI-EG* minimum temperature humidity index for each day of the period

^b^ *AFC* Age at first calving, *IFL* Interval from the first to last service, *CR* Conception rate of first service

^c^ *S1* Reference period, *S2* periods selected based on the Akaike’s information criterion

^d^ the number of overlapping animals in the top sires in the 1 and 99%, 5 and 95%, 10 and 90%, and 25 and 75% quantiles of EGs

**Fig. 4:**
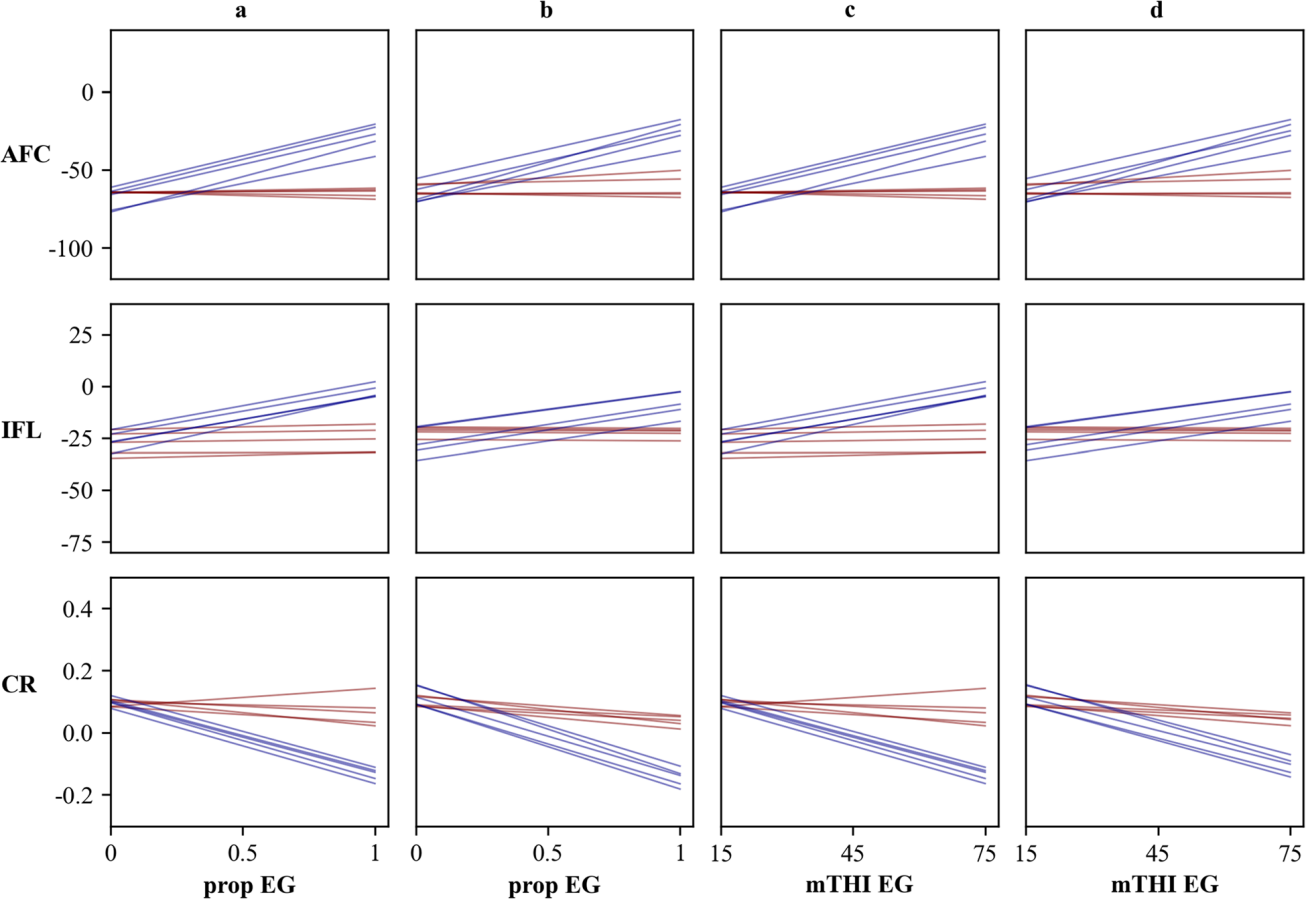
The re-ranking plots for gEBVs of sires. The blue and red lines represent sensitive and resilient sires, respectively. **a** Re-ranking plots for three traits estimated using prop-EG under S1. The x-axis is the proportion of days exceeding the threshold with a range of 0 to 1 and y-axis is gEBV of sire. **b** Re-ranking plots for three traits estimated using prop-EG under S2. The x-axis is the proportion of days exceeding the threshold with a range of 0 to 1 and y-axis is gEBV. **c** Re-ranking plots for three traits estimated using mTHI-EG under S1. The x-axis is the minimum THI with a range of 15 to 75 and y-axis is gEBV. **d** Re-ranking plots for three traits estimated using mTHI-EG under S2. The x-axis is the minimum THI with a range of 15 to 75 and y-axis is gEBV

We further visualized breeding value re-ranking by plotting gEBV of sires with the most preferential intercepts (gEBV less than average minus two times standard deviation for AFC and IFL; gEBV greater than average plus two times standard deviation for CR) in Fig. 4. The top 5 sires with the flattest slopes (more climatic resilient) were drawn in red, while the top 5 sires with the steepest slopes (more climatic sensitive) were drawn in blue. In this case, sires that are sensitive to the environments (blue lines), would perform worse than those with flat slopes (red lines) under heat stress conditions. For instance, the gEBV of CR is 0.10 when prop-EG is 0, but the gEBVs for blue lines decreased to around − 0.15 when prop-EG is 1. Meanwhile, the gEBVs of the red lines were stable along the whole prop-EG (Fig. 4a). This further verified the existence of G × E regarding the change of mTHI-EG and/or prop-EG. Larger changes were observed for gEBVs when using mTHI-EG. Implementing mTHI-EG, gEBVs of IFL for two bulls increased from around − 50 day in thermoneutral condition to 0 day in heat stress condition (Fig. 4c-d), which is nearly twice the change as gEBVs using prop-EG.

### Single-step genome-wide association analyses

**Fig. 5:**
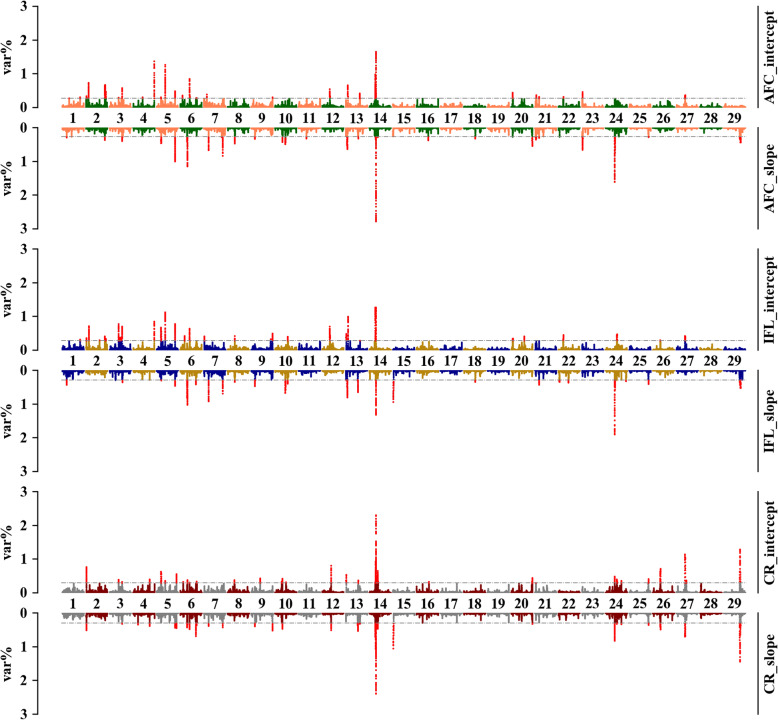
Percentage of the intercept and slope genetic variances explained by a sliding window of 20 SNPs for three fertility traits, which were estimated under scenario one of prop-EG. The x-axis is autosome segments; the y-axis represents the proportion of explained variances; the grey horizontal lines are thresholds (top 0.5%) for candidate genomic regions; and different color sets for the less relevant genomic markers indicate different traits

**Fig. 6:**
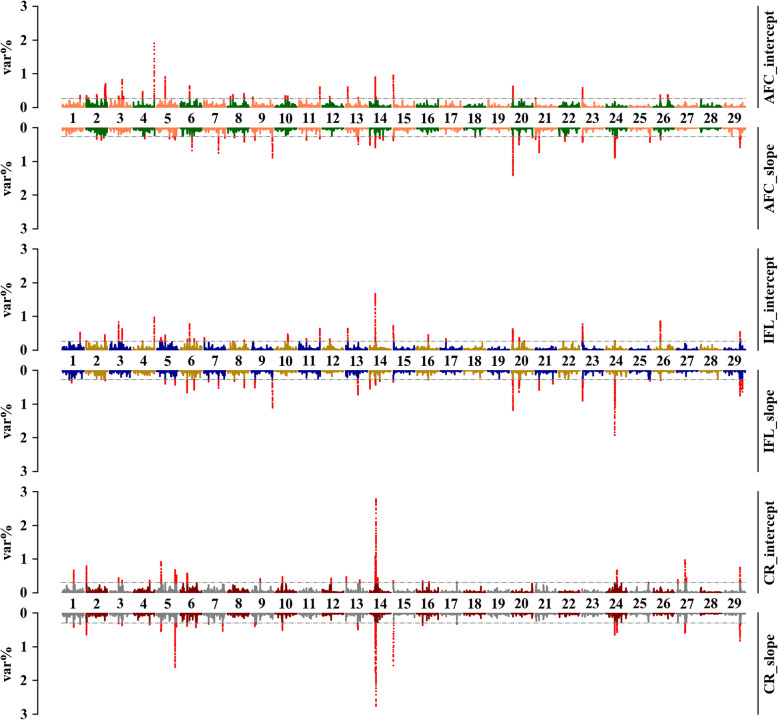
Percentages of the intercept and slope genetic variances explained by a sliding window of 20 SNPs for three traits, which were estimated under scenario two of prop-EG. The x-axis is autosome segments; the y-axis represents the proportion of explained variances; the grey horizontal lines are thresholds (top 0.5%) for candidate genomic regions; and different color sets for the less relevant genomic markers indicate different traits

Overall, similar genomic regions were detected to be associated with the same trait when using two scenarios of prop-EG, especially for CR (Figs. 5 and 6). For S1, nine regions were shared for both the intercept and the slope for AFC, among which two (from 26,669,442 to 26,802,092 and from 26,803,676 to 26,880,091 bp) were located in BTA14 and three regions (from 24,762,252 to 25,487,353 bp, from 106,901,044 to 106,946,812 bp, and from 106,948,226 to 106,980,536 bp) in BTA5, respectively. The overlapping region that explained the highest average variance (0.92% for the intercept and 2.30% for the slope) was in BTA14 (from 26,803,676 to 26,927,342 bp). Similarly, the same region (from 26,821,555 to 26,899,089 bp), which is one of the four shared genomic windows, explained 1.12 and 0.91% genetic variance for the intercept and slope of IFL, respectively. For CR, 17 regions were in common when using THI or prop-EG variables in RNM, and a narrower region (from 26,819,709 to 26,888,221 bp) in BTA14, which explained 1.83 and 1.72% genetic variance for the intercept and slope, respectively, was located in the same region detected in AFC and IFL.

The genomic windows explaining the highest variance were not connected for AFC and IFL under S2. However, the genomic region from 26,819,709 to 26,887,021 bp that explained the highest proportion of the total additive genetic variance (2.38 and 2.29% for the intercept and slope, respectively) for CR, was still located in BTA14. We detected 21 overlapping genomic windows for CR between two variables, which is more than detected for AFC and IFL (4 and 13, respectively).

More shared genomic regions were detected when the same variables (the intercept or slope) of the two scenarios were tested. For AFC and IFL, more than 10 genomic areas were connected, although they did not explain the largest amount of the total additive genetic variance. However, the longest shared region in BTA14 was still detected for both the intercept (from 26,819,709 to 26,887,021 bp) and the slope (from 26,821,555 to 26,888,221 bp) for CR. Similarly, more than 25 overlapping genomic regions were mapped for each variable of CR.

The Manhattan plots of mTHI-EG are provided in Supp. Files 7 and 8. Basically, more shared regions were mapped when using mTHI-EG compared to prop-EG, but the most associated genomic regions for each trait were found to be distributed across different chromosomes. Detailed information for genomic regions is listed in Supp. Files 9 and 10.

The mapped positional candidate genes are shown in Table 6 and Supp. Files 9 and 10. Candidate genomic regions of the intercept term were previously linked to several types of quantitative trait loci (QTL) such as milk kappa-casein percentage, metabolic body weight, average daily gain, length of productive life, dry-matter intake, conception rate, and pregnancy rate (Supp. Files 9 and 10). Most of the mapped QTLs are associated with production traits, and the rest are associated with reproduction, health, and meat/carcass traits. The identified biological processes (*P* < 0.05) related to heifer reproduction were: developmental process involved in reproduction, oocyte maturation, oocyte development, oocyte differentiation, oogenesis, placenta blood vessel development, and embryo development. Two identified pathways were related to stress response: regulation of response to stress and response to oxidative stress. Other pathways included muscle and body development.

**Table 6:** Candidate genes and QTLs for the top genomic regions of two environmental gradients (EG)

EG^a^	Trait^b^	Parameter	Chromosome	Genes	Var%	#QTL	QTL traits
Prop-EG	AFC	slope	BTA14	*CLVS1*	2.30	1	Milk unglycosylated kappa-casein percentage
	CR	intercept	BTA14	*CLVS1*	2.29	3	Milk kappa-casein percentage
	CR	slope	BTA14	*CLVS1*	1.83	1	Milk kappa-casein percentage
	CR	intercept	BTA14	*CLVS1*	1.72	2	Milk kappa-casein percentage
	AFC	slope	BTA14	*LOC112449637*	1.49	1	Milk unglycosylated kappa-casein percentage
	CR	slope	BTA5	*FKBP4, DDX11*	1.20	3	Bovine tuberculosis susceptibility, Milk kappa-casein percentage
mTHI-EG	AFC	intercept	BTA14	*CLVS1*	2.27	1	Milk glycosylated kappa-casein percentage
	AFC	slope	BTA14	*CLVS1*	2.03	1	Milk unglycosylated kappa-casein percentage
	AFC	slope	BTA14	*LOC112449637*	1.80	1	Milk unglycosylated kappa-casein percentage
	CR	intercept	BTA14	*CLVS1*	1.39	3	Milk kappa-casein percentage
	CR	slope	BTA5	*FKBP4, DDX11*	1.35	2	Milk kappa-casein percentage
	CR	slope	BTA14	*CLVS1*	1.34	3	Milk kappa-casein percentage

^a^ *prop-EG* the number of days that exceeding the threshold temperature humidity index in the period, *mTHI-EG* Minimum temperature humidity index for each day of the period

^b^ *AFC* Age at first calving, *IFL* Interval from the first to last service, *CR* Conception rate of first service

Candidate genomic regions of the slope term have been previously linked to a variety of trait groups, including luteal activity, body weight, stillbirth, and many milk-related QTLs. Similarly, most of the QTLs identified were associated with milk production traits as most of the QTLs overlapped between the two genetic terms. The reproductive biological processes identified (P < 0.05) using the slope term were: reproductive processes, fertilization, sexual reproduction, granulosa cell differentiation, oocyte development, acrosome reaction, oocyte differentiation, and regulation of luteinizing hormone secretion (Supp. Files 9 and 10). Additionally, more potential stress-related pathways were identified such as response to abiotic stimulus, detection of stimulus involved in sensory perception, response to temperature stimulus, response to radiation, negative regulation of saliva secretion, aerobic respiration, and energy derivation by oxidation of organic compounds.

### Cluster analysis for SNP effect trajectories

**Fig. 7:**
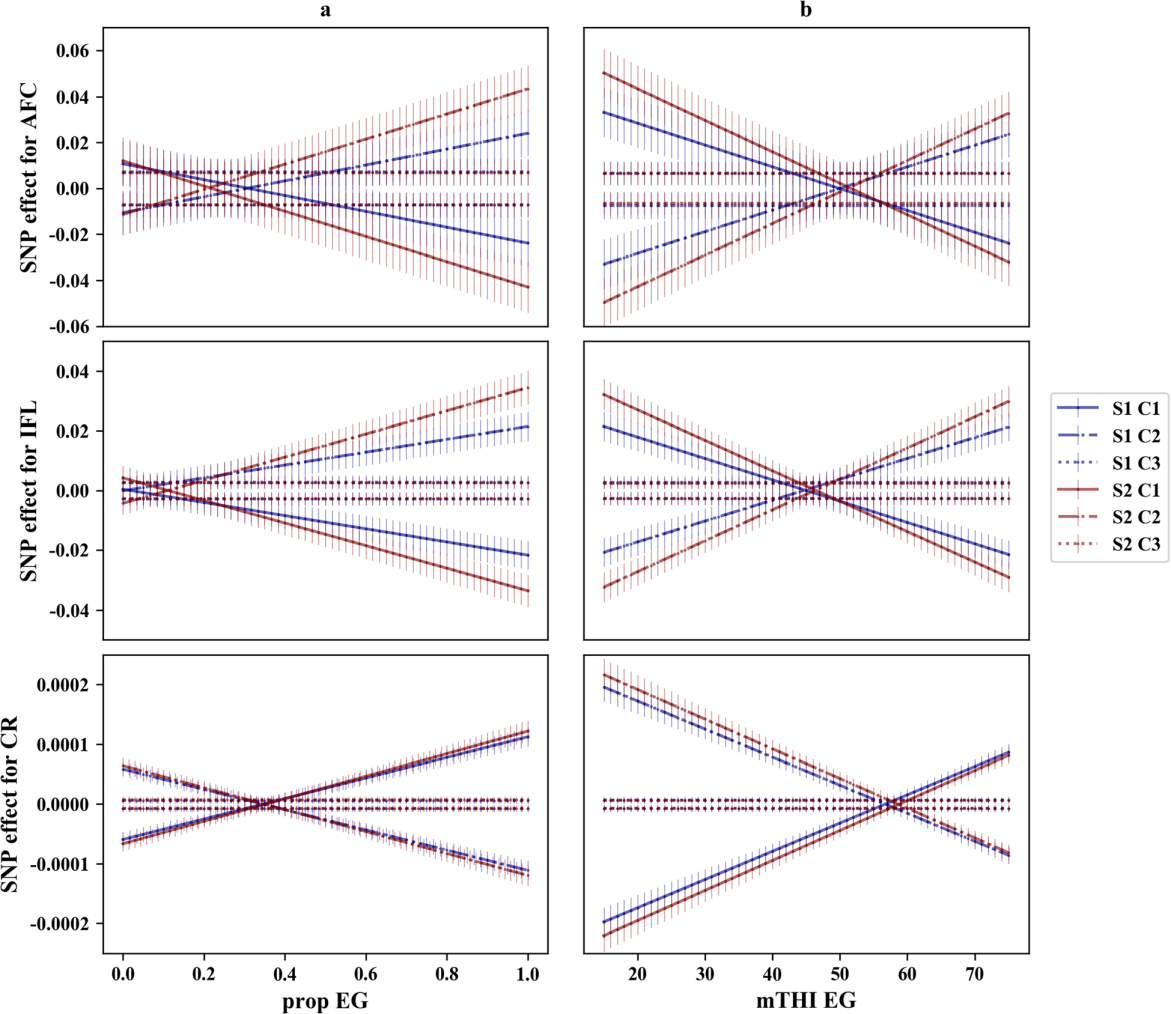
Trajectories of SNP effects changing over EGs. The x-axis is environmental gradient; the y-axis represents the SNP effects; the vertical bar is the standard deviations of SNP effects at each level of EG; the blue lines indicate scenario one; red lines indicate scenario two; and, different color sets indicate different clusters. **a** Trajectories of SNP effects changing over prop-EG. The x-axis is the proportion of days exceeding the threshold with a range of 0 to 1. **b** Trajectories of SNP effects changing over mTHI-EG. The x-axis is the minimum THI with a range of 15 to 75

The pattern of SNP effects over different EGs of each trait, scenario, and cluster (C1, C2 or C3) are shown in Fig. 7. The SNP effects remained at a specific level within each trait, and the effects for CR were almost 100 times less than those for AFC and IFL. The magnitude of SNP effects changes were higher for S2 (red lines) in all traits and EGs in C1 and C2, whereas the SNP effects in C3 were similar in each trait and scenario. The cross point of different clusters appeared later when mTHI-EG was used. Furthermore, lower standard deviations were observed for CR in different scenarios and clusters.

**Fig. 8:**
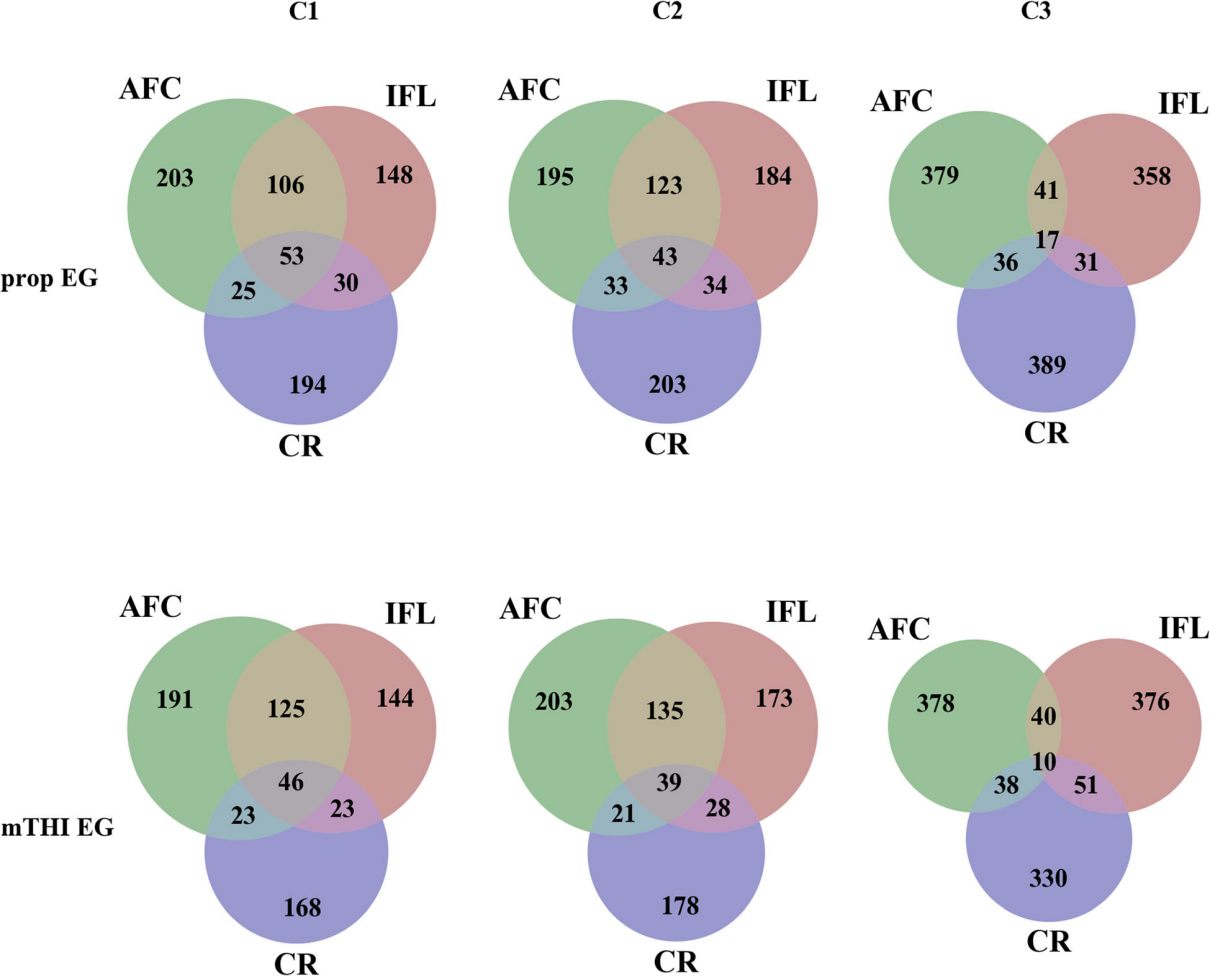
Number of shared candidate genes for each EG in different traits and clusters. C1 = SNP effects changes in preferential ways (decrease for AFC and IFL; increase for CR); C2 = SNP effects changes in the opposite ways (increase for AFC and IFL; decrease for CR); C3 = constant SNP effects over time

Approximately 50 common genes were identified in C1 and C2, whereas 10 or 17 shared genes were detected in C3. However, over 330 positional genes were detected for each trait in C3, which is nearly twice the number of genes mapped in C1 and C2 (Fig. 8). Additionally, the number of common genes between AFC and IFL was found to be higher than those shared with CR in C1 and C2. A total of 149 common genes were identified in different clusters, among which 50 genes overlapped between prop-EG and mTHI-EG.

## Discussion

### Heritabilities estimated from the conventional animal model

The heritability estimates (SE) were 0.03 (0.005) for CR, 0.06 (0.007) for IFL, and 0.16 (0.011) for AFC (Table 1). These results indicate that fertility can be improved through direct genetic and genomic selection. The AFC heritability estimate is in agreement with the literature (0.07–0.24) [23]. For heifer IFL and CR, the heritabilities observed agree with earlier studies in Irish Holstein [23] and in the same Chinese Holstein populations [4]. The current study showed that heritability estimates for interval traits (AFC and IFL) were generally higher than for the binary trait (CR), which is also consistent with several studies [7, 24, 25].

### The influence of heat stress on heifer fertility traits

Several studies have demonstrated that heat stress may have adverse effects on heifer fertility in two aspects [26]: (1) follicular development or estrus, and (2) pregnancy. The percentage of Holstein heifers having two follicular waves in the heat-stressed group (33 °C, 60% relative humidity) and thermoneutral group (21 °C, 60% relative humidity) have been shown to be 18 and 91%, respectively; the day of functional luteolysis was delayed for almost 9 days in the heat-stressed group compared to the controlled group [27]. Sakatani et al. [28] found that the estrus detection rate of non-lactating Japanese Black cows was significantly lower in summer, whereas another study of lactating Holsteins [14] also illustrated that the estrus detection rate decreased when THI exceeded 72. Follicular development and estrus detection are negatively affected by heat stress, which extends AFC and IFL. Another analysis of 20,606 cows also showed that pregnancy rates declined when THI was greater than 72, with a decrease in pregnancy rate of 1.03% per unit increase of THI [29]. In the current study, a dramatic decrease in the month of July was observed for the CR phenotypes (Suppl. File 11), which is consistent with the results of previous studies.

The critical periods found under S2 of CR, on the basis of first insemination day, were (− 10, 60) and (− 10, 30) for prop-EG and mTHI-EG, respectively, which are similar to the values reported in previous studies. Khan et al. [17] explored the effects of heat stress on the pregnancy rate using 1100 crossbred dairy cows in India and reported that pregnancy rates decreased significantly when cows experienced high THI (> 72) within a period of at least 30 days before and after the insemination day. For AFC and IFL, the critical period was earlier before the first service: (− 90, − 30) or (− 90, − 60). This may be partly due to the delay of first insemination; that is, follicular development or estrus detection may be compromised by heat stress. A study evaluating the whole cycle of estrus showed that heat stress inhibits the development of the dominant follicle during preovulatory period in heifers [27]. Thus, the onset of estrus for heifers would be delayed to the next circle due to the delayed development of follicles, and heat stress actually impacts the heifers earlier before the first insemination. Another possible explanation is that heat stress may affect heifer puberty. The age at puberty is generally around 13 months, and the average age at first service in this study was 491.95 days (around 16 months) with a standard error of 72.48 days. This may explain the reason that the boundary of the critical periods for AFC and IFL was found to be 90 days (3 months) before the first insemination. However, additional studies are needed to validate this relationship.

To the best of our knowledge, the critical periods of heat stress for heifer reproduction traits have not been well defined, and most studies focused solely on pregnancy rate or CR. Amundson et al. [30] detected negative associations of THI with pregnancy rate for *Bos taurus* crossbred cows in all three breeding periods: (0, 21), (0, 42), and (0, 60). Another study indicated that a high heat load 3–5 weeks before and 1 week after service was associated with reduced CR in cattle [16]. In Schüller et al. [18], the CR of lactating dairy cows was negatively affected by heat stress in both before and after the day of insemination (− 42, 31), with the greatest negative impact in (− 21, − 1). Li et al. [31] proposed a window search algorithm to detect critical photothermal time for flowering in plants, which is assessed by the Pearson correlation coefficients of average photothermal time and the population means of flowering time. However, all the time combinations are not significantly (with the absolute value of coefficients less than 0.05) correlated with target traits when this approach is utilized in the current study. This may be due to the low heritable feature of female fertility traits in cattle, indicating that the phenotypes are influenced by complex factors. Thus, the impact of THI on fertility phenotypes could be nonlinear or relatively smaller. The results of the current research indicated that the critical period of heat stress is trait-related, and the periods we identified for CR are within the range reported in previous studies, which provides substantial support for the results of AFC and IFL.

The overlapping test of the two EGs under two scenarios (Table 3) indicated that prop-EG and mTHI-EG capture different mechanisms. For instance, prop-EG equaled 0.5 in a period of 60 days when the THI of any 30 days in this period exceeded the threshold. However, the average daily THI of these 30 days may be 73 or 78 (more severe heat stress). Similarly, prop-EG equaled 0 when THI did not exceed the threshold for all the days in a period, but these days may occur in autumn or winter (cold stress). Consequently, prop-EG could not assess cold or severe heat conditions as mTHI-EG; it represents the average heat load of heifers, i.e., the proportion of days experiencing heat stress conditions, which would not include all the climatic variation due to the use of a THI threshold. CR may be more sensitive to climatic changes in comparison to AFC and IFL as it resulted in fewer common animals selected across the two EGs.

### Variance components estimated from RNMs and G × E

We estimated variance components using RNMs with the matrices ***A*** and ***H*** (Table 4 and Supp. File 4). The genetic variances obtained from conventional RNMs and those from genomic RNMs were similar for all traits and agree with the results of previous studies (e.g., [7, 32]). Heritabilities estimated from RNMs were generally at similar levels, compared with those estimated from conventional animal models (Fig. 2). Zhang et al. [7] detected heterogeneities in heritabilities across different EGs for fertility traits based on both matrices, which is consistent with our study. A recent study found the genetic variances of production traits varied at different THI levels, and a quadratic curve was observed in the heritabilities of protein yield [8]. In Danish Holstein populations, heterogeneities of heritabilities of several fertility traits were observed across different production levels or grass ratio in feed [6, 33]. Although heritabilities vary across EGs in different analyses, patterns of heritabilities with changes in the environment are predictable. Various environmental indicators and analyzed traits could account for this result. Heterogeneities in heritabilities provide the insight that future genetic evaluations should consider different environmental variances to obtain accurate results. Incorporating heat stress into breeding objectives could be helpful for the correct selection of animals in different environments, especially in countries where climatic conditions are highly variable.

Based on the t-test for the variance of the slope, a statistically significant G × E was observed for all traits in this study [5]. Moderate to strong negative correlations were detected between the intercept and slope for each trait. This negative relationship was also found by other researchers who estimated the variance components of cow fertility traits using RNM [6, 7]. Previous studies have suggested that a low correlation between the intercept and slope could indicate re-ranking of animals across different environments [34, 35]. In addition, Liu et al. [6] illustrated that this negative correlation could increase the magnitude of G × E. Collectively, robust animals with preferential intercepts and flat slopes are able to perform well across various environments [36]. Based on RNMs, individual breeding values for each THI condition can be appropriately estimated to enable selection of heat tolerant cows. These estimates could be more accurate for bulls whose daughters have different records under different environments. Genetic correlations between different EGs estimated using conventional and genomic RNMs were similar (Fig. 3 and Supp. File 5). The more divergent EGs were less correlated, which is consistent with studies using different EGs [6, 7, 37]. This indicates that the re-ranking of sires may occur in different EGs, as indicated in Table 5. The re-ranking was more obvious for CR, in comparison to the other two traits, indicating that CR has a higher sensitivity under different THI. As mentioned in the previous section, heat stress has adverse effects on follicular development, which is directly associated with conception. Furthermore, we observed that the re-ranking was stronger when using mTHI-EG for AFC and IFL, which validates the hypothesis that mTHI-EG captured more variation in environments than prop-EG.

In a breeding scheme for improving heat tolerance, the best approach could involve selecting the best-performing individuals in heat stress conditions, provided that they are not underperforming in thermoneutral conditions. These robust animals can be presented graphically as shown in Fig. 4. For these traits, all animals have consistent intercepts, whereas the gEBVs of heat-tolerant heifers (red lines) did not change dramatically along all environmental conditions. Lower AFC and IFL are linked to greater farm profit, and thus, are more desirable. All the robust heifers performed better in higher THI conditions. Adverse trends were observed for CR because a higher CR is preferential. The magnitude of re-ranking was higher when using mTHI-EG. This is additional evidence that mTHI-EG is more sensitive than prop-EG. The use of genomic selection is a promising route for implementing selection schemes for improve heat tolerance.

### Candidate genomic regions for the intercepts and slopes

We performed ssGWAS to detect candidate genomic regions associated with heifer fertility and heat sensitivity (Figs. 5 and 6, Supp. Files 7 and 8). Some identified genomic regions are common between two traits or two variables in the same trait (Supp. Files 9 and 10). In general, most of these genomic regions explained a small (< 1%) proportion of the total additive genetic variance, indicating that fertility and heat tolerance are largely polygenic traits. However, the functional analysis confirmed that the fertility traits are influenced by heat stress. For example, some of the genomic regions were previously reported to be associated with pathways such as response to abiotic stimulus, detection of stimulus involved in sensory perception, response to temperature stimulus, response to radiation, negative regulation of saliva secretion, and aerobic respiration and energy.

As expected, the genomic regions associated with the intercept (average performance in thermoneutral conditions) were linked with several reproductive genes. For example, the overlaps of genomic windows between the intercept of AFC and IFL contained genes such as *AMHR2*, *SP1*, *KRT8*, and *KRT18*. Ilha et al. [38] found that the mRNA expression levels of *AMHR2* decreased in the follicles during follicular deviation, whereas another study also indicated that *AMHR2* plays a role in follicular development by regulating granulosa cells [39]. *KRT18* may be a molecular marker for bovine microfold cells in the follicle-associated epithelium [40]. Together with *KRT8*, these two keratin family genes influence the bovine estrous cycle with regards to luteal cells [41]. *SP1* has been demonstrated to co-express with other regulators to control early placental differentiation [42]. *PLAG1*, which was mapped in the most evident region of BTA14 for AFC and IFL, has been often reported to be associated with growth and reproduction traits in cattle [43–45]. The overlapping genomic regions between AFC and CR contained other growth-related genes such as *SDC3* [46] and *FABP3* [47]. One possible explanation is that most heifers are physiologically immature at the time of first insemination.

Various candidate genes associated with heat tolerance (slope term) were also reported in the literature. Several candidate genes are related to cow reproduction such as *GPER1*, which has been reported to induce the non-genomic suppression luteinizing hormone secretion in cattle [48] and was mapped in both AFC and IFL in the genomic region of BTA25. *RAD51* has been reported to be associated with bovine oocytes meiosis progress [49, 50]. Some candidate genes (e.g., *LAP3*, *GLYCAM1*, *PDE1B*, *MICALL2*, *NPC1*) are related with other economically-important traits in cattle such as milk production, carcass traits, body weight, body height, and body length [51–55]. Additionally, several genes were annotated to be associated with stress response in cattle. *HCRTR1*, which regulates orexin receptor type 1, has been suggested to participate in negative feedback regulation in the adrenal gland [56]. Several studies examined cultured bovine adrenal cells and indicated that the products of *AGRP* would inhibit the cortisol production of adrenal gland [57, 58]. The stress reaction of animals activates the hypothalamic-pituitary-adrenal axis, together with an increase in the cortisol concentration [59]. Thus, *HCRTR1* and *AGRP* may play vital roles in the cattle stress reaction process. White et al. [60] reported that endogenous *PC* expression in bovine primary hepatocytes and kidney epithelial was significantly higher in thermal stress conditions, which indicate that *PC* may contribute to the physiological response to thermal stress. *PC* has been reported to be associated with fatty acids regulations while feeding and thus affect the feed intake of cattle [60, 61].

The biological processes identified in functional analyses were mainly reproduction- and stress response-related, which corroborates with our findings. The QTLs identified in this study are mainly related to milk production traits (Supp. Files 9 and 10), indicating that these economic traits may change as the climate conditions become more extreme. Most of the QTLs overlapped when using different EGs, and many were located in BTA14. Costa et al. [62] reported that the QTLs in BTA14 (24.3 Mb) and BTA24 (23.4 Mb) are associated with AFC in Nellore. The genomic regions detected from 23 to 26 Mb in BTA14 and BTA24 were most evident in the Manhattan plots among the different traits and EGs. Mota et al. [45] supported that the region of BTA14 plays a key role in heifer puberty through growth hormone signaling and may be regulated by *PLAG1*. Another genomic region with high genetic variance is located in BTA29 (from 44.3 to 44.9 Mb), which includes the heat stress-related gene *PC*. Most of the QTLs were shared by the intercept and slope, which is consistent with the strong negative genetic correlations between them. The fertility-related QTLs would be evident in heat stress conditions as well because the change in environments may alter the genetic expression of these regions when a strong G × E exists.

### Clustered SNP effects and genes

The trajectory of SNP effects over EGs (Fig. 7) suggests substantial SNP by environment interactions, which has also been reported for reproduction traits in pigs [32] and cattle [45]. The dramatic changes in C1 and C2 in S2 indicates that the critical periods, which were chosen through AIC, may capture more genetic variation. As for CR, half of the periods overlapped in S1 (− 30, 30) and S2 (− 10, 30 or 60), which may explain the similarity in the trajectories between the two scenarios. For C1 and C2, the variation in SNP effects was greater at high levels of prop-EG (Fig. 7a) than at low or middle levels, which was expected due to the higher genetic variance detected at high prop-EG levels compared to low and middle prop-EG levels (Fig. 2a). Similarly, this explains the greater SNP effects at both high and low levels of mTHI-EG and the late “cross point”, which represents the lowest genetic variance at middle mTHI-EG levels (Fig. 2b). These findings agree with those of Silva et al. [32]. The effects of top SNPs were more constant (lower standard deviation) in CR compared with AFC and IFL. This finding may be due to the relatively low heritability of CR, which causes smaller effect variations in each level of EG.

Three SNP clusters were inspected to identify common genes among all the fertility traits (Fig. 8). As expected, more candidate genes were shared between interval traits (AFC and IFL), especially in C1 and C2. The number of overlapping genes among all the traits and EGs were comparable for each cluster. For C3, the top SNPs with slope effects close to 0 were chosen, but the variation was negligible, i.e. SNP effects of the slope ranged from 9.48e-9 to 1.47E-11 for all traits. Thus, a slight change in the effect estimates may cause re-ranking of SNPs, which results in a small proportion of shared genes among traits in C3. The trajectories of C1 and C2 (Fig. 7) indicate the existence of SNP by environment interactions, and therefore, some related genes may be activated at specific temperature and humidity levels. Thus, candidate genes that play an important role in SNP effect changes (C1 and C2) are the priority of the current research.

Some genes detected in both EGs were reproduction– or milk–related. For instance, *MLH1* is associated with oocyte development [50] and *EOMES* plays a vital role in the early pregnancy stage of ruminants. Sakurai et al. [63] reported that cattle *EOMES* expression increases when conceptuses attach to the uterine epithelium. In a Chinese Holstein population, Han et al. [64] profiled the genetic effect of *ACACB*, which affects milk composition traits, using whole genome re-sequenced data. As production and reproduction traits are genetically related [2], it is reasonable that some production associated genes were also detected in this study.

When prop-EG was considered, reproductive genes *NR5A2*, *THBS2* and *PRKCE* were identified. *NR5A2* was mapped in C1, and it has been reported to affected steroidogenic pathways of progesterone production during the luteal phase of the estrous cycle in cattle [65]. *THBS2* and *PRKCE* contain several candidate SNPs in C2, and their potential luteolytic functions were illustrated in previous studies [66, 67]. Additionally, *EGFR*, whose expression is related to bovine reproduction stage [68], was detected in both C1 and C2. Wijayagunawardane et al. [69] explored the potential mechanisms responsible for the detrimental effect of heat stress by exposing bovine oviductal epithelial cells to heat stress conditions (40 and 43 °C). The results indicated that *EGFR* could be involved in the regulation of the bovine oviductal microenvironment, but these regulatory mechanisms may be compromised in the presence of heat stress. This indicates that the regulatory functions of detected reproductive (or even milk- and growth-related) genes might be compromised in heat stress conditions. Thus, these altered SNP effects were observed at higher levels of prop- and mTHI-EGs (Fig. 7).

When mTHI-EG was used, *GUCY1B1* was identified in C2. Several papers [70, 71] have demonstrated that *GUCY1B1* interacts with heat shock protein 90 (*HSP90*), whereas Khan et al. [72] detected *HSPA13* as a differentially expressed gene in heat-stressed bovine granulosa cells. This indicates that the interaction between *GUCY1B* and *HSP90* in cattle may be related to the heat stress response.

## Conclusions

We analyzed the impact of heat stress on dairy cattle based on three fertility traits. The critical periods, which are when heifers may be more affected by heat stress, were found to be related to the environmental gradient used, centered on the first insemination day. This indicates that detailed analysis for other traits should be applied to derive this period. Genetic parameters suggest significant and considerable magnitude of G × E for all three heifer fertility traits, indicating that breeding values may change under heat stress conditions for these traits. The re-ranking of sires between different environments further demonstrates the effects of G × E on animal breeding. Several reproduction–, growth–, production–, and resilience–related genes and QTLs were identified in the candidate genomic regions affecting fertility traits. Overall, G × E models should be integrated into current dairy cattle breeding schemes to select more climatic resilient animals. The heat stress-related genes or QTLs are important for exploring the mechanisms of heat stress response in dairy cattle.

## Materials and methods

### Data

Field records of birth, service and calving from 2005 to 2018 for heifers raised in 15 Holstein cattle farms (Sunlon Livestock Development Co. Ltd) in Beijing, China, were collected through the herd management software AfiFarm (AfiFarm, www.afimilk.com.cn). All herds were kept in a free-stall design and included 1000 to 2000 heifers and the management strategies in these farms are similar. The analyzed heifer traits are AFC (age at first calving, days), IFL (interval from the first to last service, days), and CR (conception rate of first service). CR was coded as 1 when there was a confirmed pregnancy after the first service and 0 otherwise. The IFL was 0 when a heifer was pregnant after the first service. Further criteria for the data editing included AFC between 500 and 1100 days and IFL between 0 and 365 days. Records with values outside these ranges were dropped of further analyses. Animals which changed herds during the analyzed period were also excluded. The number of records for each trait after editing was 56,998 (Table 1). The pedigree was derived from field birth records and each animal was traced back at least three generations, as suggested by previous studies [6, 7]. The final pedigree contains 181,693 individuals, among which 6556 are sires.

Phenotypes (of daughters) and genotypes were available for 3731 heifers and 537 bulls. All bulls and a subset of 2379 heifers were genotyped using the GeneSeek Genomic Profiler Bovine 50 K Chip, whereas the remaining 1352 heifers were genotyped using the GeneSeek Genomic Profiler Bovine 150 K Chip. In addition, 1769 heifers genotyped with the 150 K chip were included in the reference population to improve imputation accuracy. The animals genotyped with the 50 K chip were imputed to the 150 K using the Beagle v5.0 software [73] with an imputation accuracy greater than 0.95. After imputation, SNP markers were filtered by removing markers with minor allele frequency lower than 0.05, missing rate greater than 0.10, and presenting extreme deviation from the Hardy–Weinberg equilibrium (*P* ≤ 10E− 5). Individuals with genotype call rate lower than 0.90 were dropped and only autosomal markers were retained for this study. After quality control, 111,068 SNPs remained in the dataset.

Hourly recorded temperature data of Beijing (all farms are within 30 km from the weather station) during the test years were obtained from the National Oceanic and Atmospheric Administration (www.noaa.gov). Hourly THI was calculated using the following formula [74]:

$$ \mathrm{THI}={\mathrm{T}}_{\mathrm{db}}+\left(0.36{\mathrm{T}}_{\mathrm{dp}}\right)+41.2 $$

where T_db_ is the hourly dry bulb temperature (°C) and T_dp_ is the dew point temperature (°C). Then, the daily THI was calculated by averaging hourly values.

### Models

Many studies have shown that the estimates from linear models for categorical fertility traits are similar to those from threshold models, while the former require less computation time [75, 76]. Furthermore, several studies managed to detect G × E for categorical fertility traits using linear RNMs [6, 7]. Thus, linear models were used for all fertility traits in the current study. Variance components were obtained using the following single-trait animal model:

$$ \mathbf{y}=\mathbf{Xb}+\mathbf{Za}+\mathbf{e} $$

where **y** is the phenotype of each heifer for the three fertility trait (AFC, IFL, and CR); **X** is an incidence matrix connecting the vector of fixed effects **b** (herd-year-month of the first service, service technician and gender-controlled semen) to **y**; **Z** is an incidence matrix connecting **a** (additive genetic effect) to **y**, and **e** is the residual effect. The following RNM was used to investigate G × E:

$$ \mathbf{y}=\mathbf{Xb}+{\mathbf{Z}}_{\mathbf{0}}{\mathbf{a}}_{\mathbf{0}}+{\mathbf{Z}}_{\mathbf{1}}{\mathbf{a}}_{\mathbf{1}}+\mathbf{e} $$

where **Z**_**0**_ is an incidence matrix connecting **a**_**0**_ (intercept) to **y**, and **Z**_**1**_ is an incidence matrix containing EGs as covariables to connect **a**_**1**_ to **y** (slope). It was assumed that $$ \left[\begin{array}{c}{\boldsymbol{a}}_0\\ {}{\boldsymbol{a}}_1\end{array}\right]\sim \boldsymbol{N}\ \left(0,\boldsymbol{K}\bigotimes \left[\begin{array}{cc}{\sigma}_{a_0}^2& {\sigma}_{a_0{a}_1}\\ {}{\sigma}_{a_0{a}_1}& {\sigma}_{a_1}^2\end{array}\right]\right) $$, where ***K*** is ***A*** (numerator relationship matrix) for pedigree-based BLUP, or ***H*** (combined pedigree-genomic relationship matrix) for ssGBLUP.

The matrix ***A*** was constructed using pedigree data only for conventional BLUP, whereas for the ssGBLUP models, the inverse of the ***H*** matrix (***H***^***− 1***^), which was calculated as follows [20, 77]:

$$ {H}^{-1}={A}^{-1}+\left[\begin{array}{cc}0& 0\\ {}0& {G}^{-1}-{A}_{22}\end{array}\right] $$

where ***A***_***22***_ is the subset of **A** for genotyped individuals and ***G*** is the blended genomic relationship matrix. ***G*** was built using (1 − ω)***G***_**0**_ + ω***A***_**22**_, where ω is the assumed weight of the genetic variance not captured by markers and was set to 0.05 according in previous studies [7, 78]. ***G***_***0***_ was constructed using the method proposed by [79]. Finally, ***G*** was tuned to have the same scale as ***A***_***22***_ [80, 81].

The random residual vector **e** is assumed to follow $\mathbf{N}\sim \left(0,\mathbf{I}{\boldsymbol{\upsigma}}_{\mathbf{e}}^{\mathbf{2}}\right)$, where **I** is an identity matrix. The residual variances were assumed to be the same across different EGs, based on the results of preliminary analyses, which suggested that residual variances were similar across different levels of EG. The full data was divided into three subsets based on the percentile of EGs (percentiles of EG of ≤0.1, 0.1–0.3, and ≥ 0.3) to achieve similar sample sizes across subsets.

The (co) variance components for RNMs were estimated using the average information REML method implemented in the BLUPF90 programs [82]. The standard errors of (co) variance components were obtained from the average information matrix. The standard errors of heritabilities for different EGs were calculated using the Taylor series expansions [83].

### Critical period and environmental gradient

To evaluate the impact of heat stress on heifer reproduction, the basic assumption was that heifers experiencing higher THI in a specific period have reduced fertility performance based on critical periods, as defined in Fig. 1. The levels of prop-EG range from 0 to 1 according to its definition. For mTHI-EG, the levels of EG are the true minimum THI value, ranging from 15 to 75. Two scenarios were included in each EG to select critical period: 1) Scenario 1 (S1) contained only one reference period of 60 days, which goes from 30 days prior to the first insemination to 30 days after the first insemination [17] for three reproductive traits and two EG schemes; scenario 2 (S2): the critical periods were selected based on the AIC of RNMs, which resulted in different critical periods for each trait or EG scheme. A total of 19 combinations were tested in S2, and periods with the lowest AIC were chosen (Supp. File 2). For each trait, two critical periods (S1 and S2) were chosen under each EG (prop-EG and mTHI-EG) to estimate the (co) variance components of the RNMs. The relationships of the two EG schemes were also evaluated by calculating the equivalent values: the proportion of days exceeding threshold THI when the average minimum THI of this period at a certain level. Afterwards, the overlapping heifers were counted for each equivalent environmental condition between two EG schemes.

### Magnitude of G × E

G × E exists if the variance of the slope was significantly different from zero by using a one-tailed t-test with the significance level of 0.05 [6, 33]. One possible consequence of G × E is the re-ranking of animals across different environments [5]; thus, the top 50 sires with at least 20 daughters having phenotypes were selected to assess the magnitude of re-ranking. Re-ranking plots were drawn to show the change pattern of breeding values for sires with the most preferential intercepts or with the lowest slopes.

### Single-step genome-wide association study (ssGWAS)

The marker effects of the intercept and the slope for all traits were estimated using the ssGWAS method proposed by Wang et al. [21]. The percentage of genetic variance explained by a moving genomic window of 20 adjacent SNPs was obtained, by applying the postGSf90 package [84]. The number of adjacent SNPs were defined based on the level of linkage disequilibrium in this population, following [7]. The top 0.5% genomic regions that explained the highest genetic variance of intercept or slope was considered as the candidate genomic regions. Subsequently, candidate genes or QTLs within the candidate genomic regions were annotated based on the ARS-UCD1.2 genome (http://hgdownload.soe.ucsc.edu/goldenPath/bosTau9/bigZips/genes/) and the Cattle QTL database (https://www.animalgenome.org/cgi-bin/QTLdb/). The biological processes of candidate genes were annotated using the PANTHER Classification System [85].

### Cluster analyses of relevant SNPs

The SNPs were chosen to investigate the trajectories of their effects. Firstly, SNPs were ranked according to the magnitude of their slope effects for each trait, EG, and scenario. Then, three clusters (C) of SNPs were obtained according to the trajectory of their SNP effects across each EG: C1 = SNP effects changes in preferential ways (decrease for AFC and IFL; increase for CR); C2 = SNP effects changes in opposite ways (increase for AFC and IFL; decrease for CR); C3 = constant SNP effects over time. For C1 and C2, the top 0.5% (*n* = 555) SNPs with the highest or the lowest slope effects were selected, whereas for C3, the 0.5% SNPs with slope effects closest to zero were selected. Choosing the top 1% SNPs for trajectory analyses has been implemented in several GWAS studies based on the 50 K SNP panel [32, 86]. The results were visualized based on the average and standard deviation of SNP effects in each EG to show the differences among the three clusters. For C3, SNPs were grouped into two categories according to their average effects (lower or higher than 0). Only the genes containing SNPs further confirmed in the cluster analysis and shared among three traits were further annotated for biological functions.

## Supplementary Information

**Additional file 1: Figure S1.** Climate conditions in Beijing, China, during experiment years.

**Additional file 2: Table S1.** Detailed results of AICs for 19 tested combinations.

**Additional file 3: Table S2.** The equivalents of prop and mTHI-EGs and top 1% overlapping heifers between these two kinds of EGs for each trait and scenario estimated by ***A*** matrix.

**Additional file 4: Table S3.** The genetic parameters estimated by ***A*** matrix.

**Additional file 5: Figure S2.** Heritabilities estimated by RNMs with the matrix ***A***.

**Additional file 6: Figure S3.** Genetic correlations estimated by RNMs with the matrix ***A***.

**Additional file 7: Figure S4.** Percentages of the intercept and slope genetic variances explained by a sliding window of 20 SNPs for three traits, which were estimated under scenario one of mTHI-EG.

**Additional file 8: Figure S5.** Percentages of the intercept and slope genetic variances explained by a sliding window of 20 SNPs for three traits, which were estimated under scenario two of mTHI-EG.

**Additional file 9: Table S4.** Detailed information for genomic regions, genes and QTLs detected using prop-EG.

**Additional file 10: Table S5.** Detailed information for genomic regions, genes and QTLs detected using mTHI-EG.

**Additional file 11: Figure S6.** Average conception rate of Holstein population in different months.

## Data Availability

The datasets used and/or analyzed during the current study are available from the corresponding author on reasonable request.

## Abbreviations

G × E: Genotype by environment interaction
QTL: Quantitative trait loci
THI: Temperature humidity index
EG: Environmental gradient
RNM: Reaction norm model
BLUP: Best Linear Unbiased Prediction
ssGBLUP: Single-step genomic BLUP
ssGWAS: Single-step genome-wide association study
AFC: Age at first calving
IFL: Interval from first to last service
CR: Conception rate
prop: The proportion of days that exceeded the threshold THI in candidate period
mTHI: Minimum THI for each day of the candidate period
AIC: Akaike Information Criteria
gEBV: Genomic estimated breeding value
S: Scenario
C: Cluster
