# Supplementary figures and images for: Genotype-by-environment interaction in Holstein heifer fertility traits using single-step genomic reaction norm models
Source: BMC Genomics. 2021 Mar 17;22:193. doi: 10.1186/s12864-021-07496-3 (PMC7968333; doi:10.1186/s12864-021-07496-3)

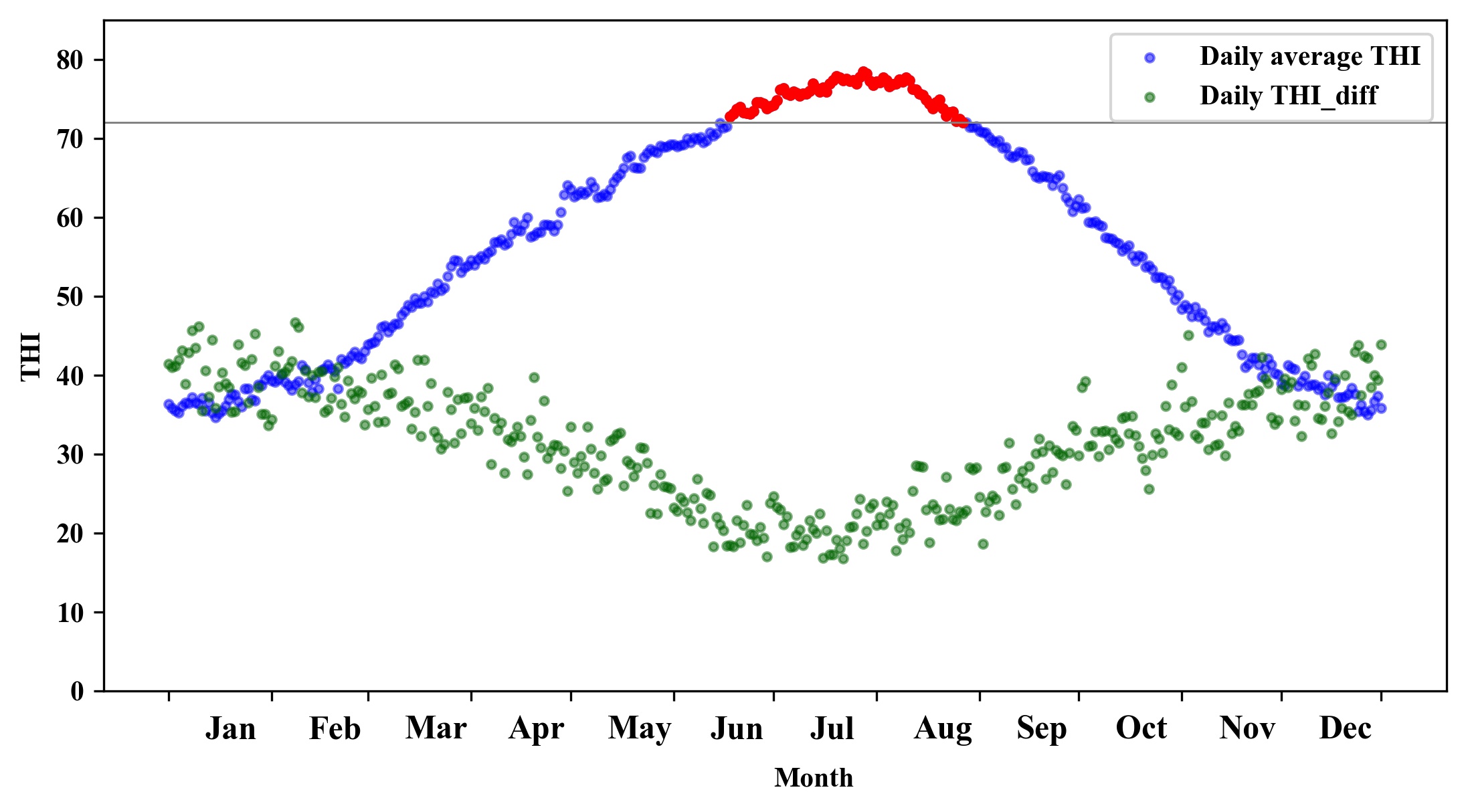

Supplement: Supplementary file 1 — Additional file 1: Figure S1. Climate conditions in Beijing, China, during experiment years. [file 12864_2021_7496_MOESM1_ESM.jpg]

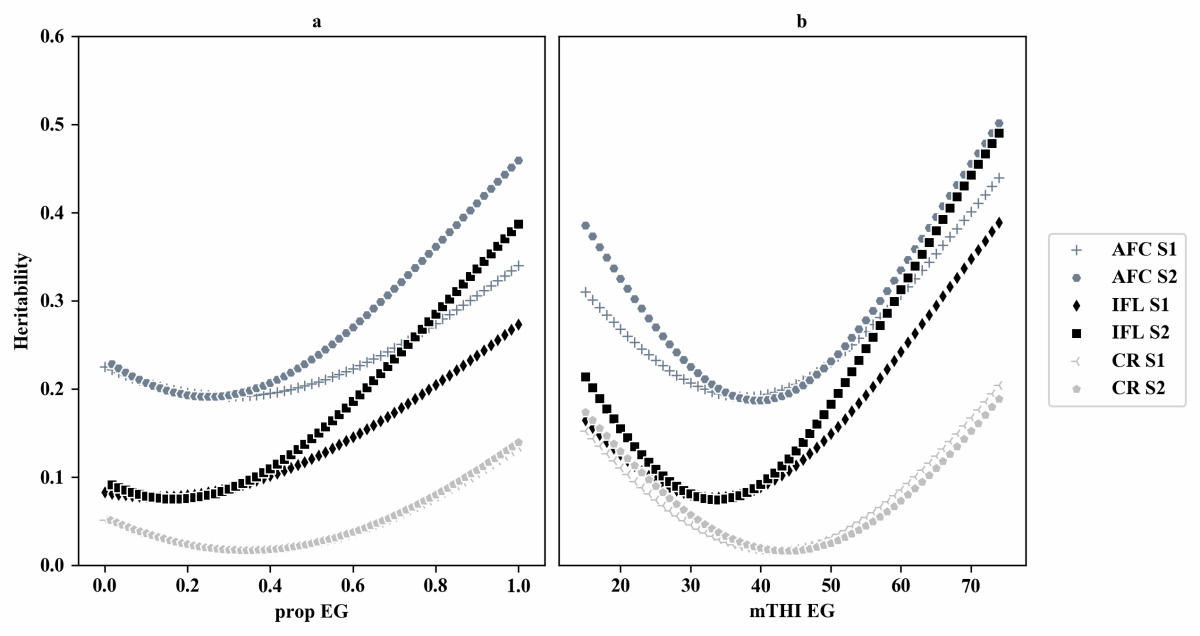

Supplement: Supplementary file 5 — Additional file 5: Figure S2. Heritabilities estimated by RNMs with the matrix A. [file 12864_2021_7496_MOESM5_ESM.jpg]

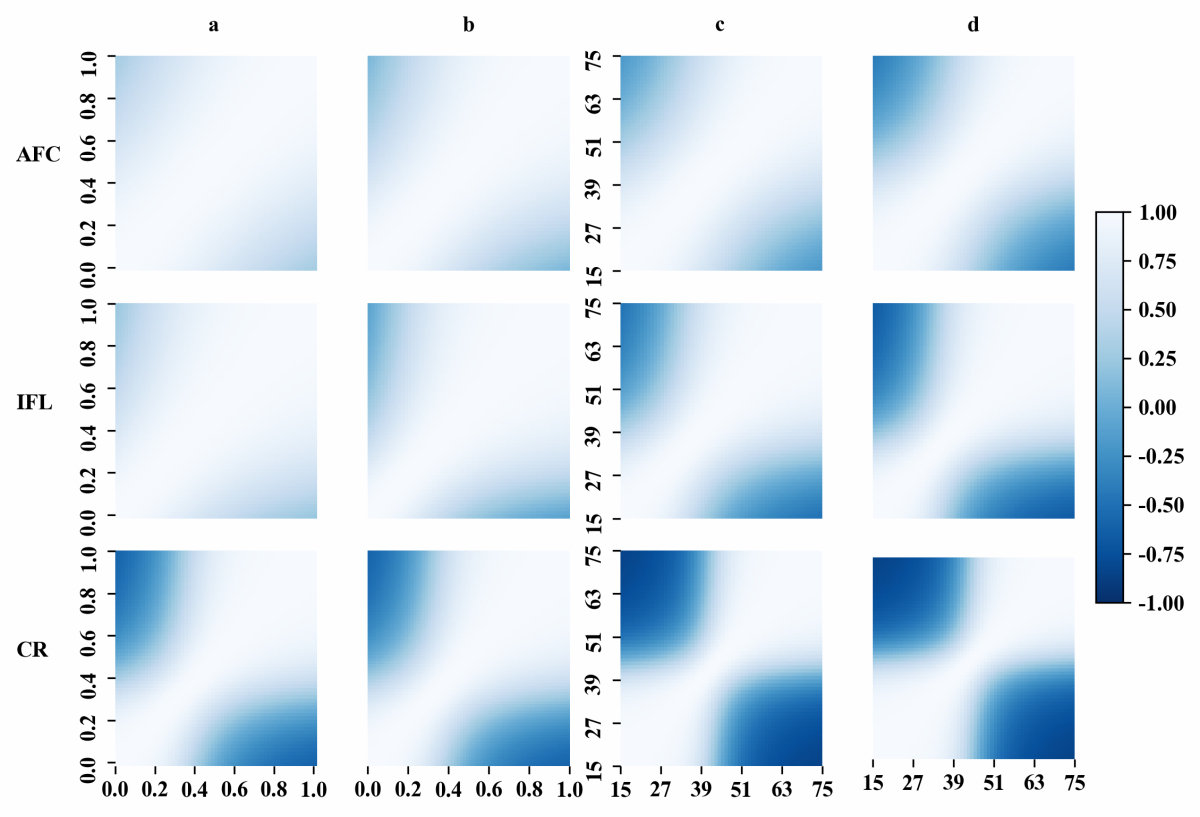

Supplement: Supplementary file 6 — Additional file 6: Figure S3. Genetic correlations estimated by RNMs with the matrix A. [file 12864_2021_7496_MOESM6_ESM.jpg]

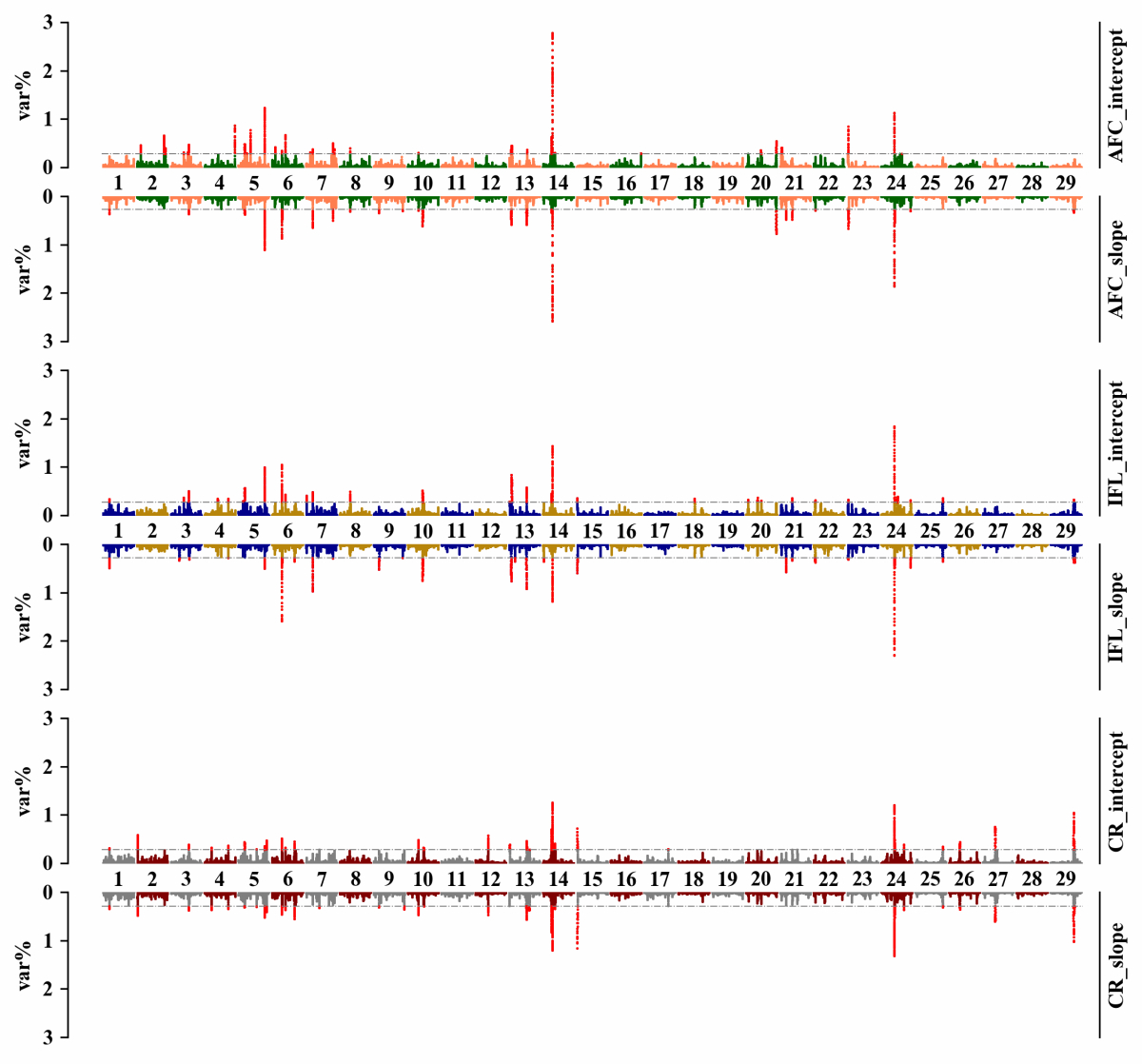

Supplement: Supplementary file 7 — Additional file 7: Figure S4. Percentages of the intercept and slope genetic variances explained by a sliding window of 20 SNPs for three traits, which were estimated under scenario one of mTHI-EG. [file 12864_2021_7496_MOESM7_ESM.jpg]

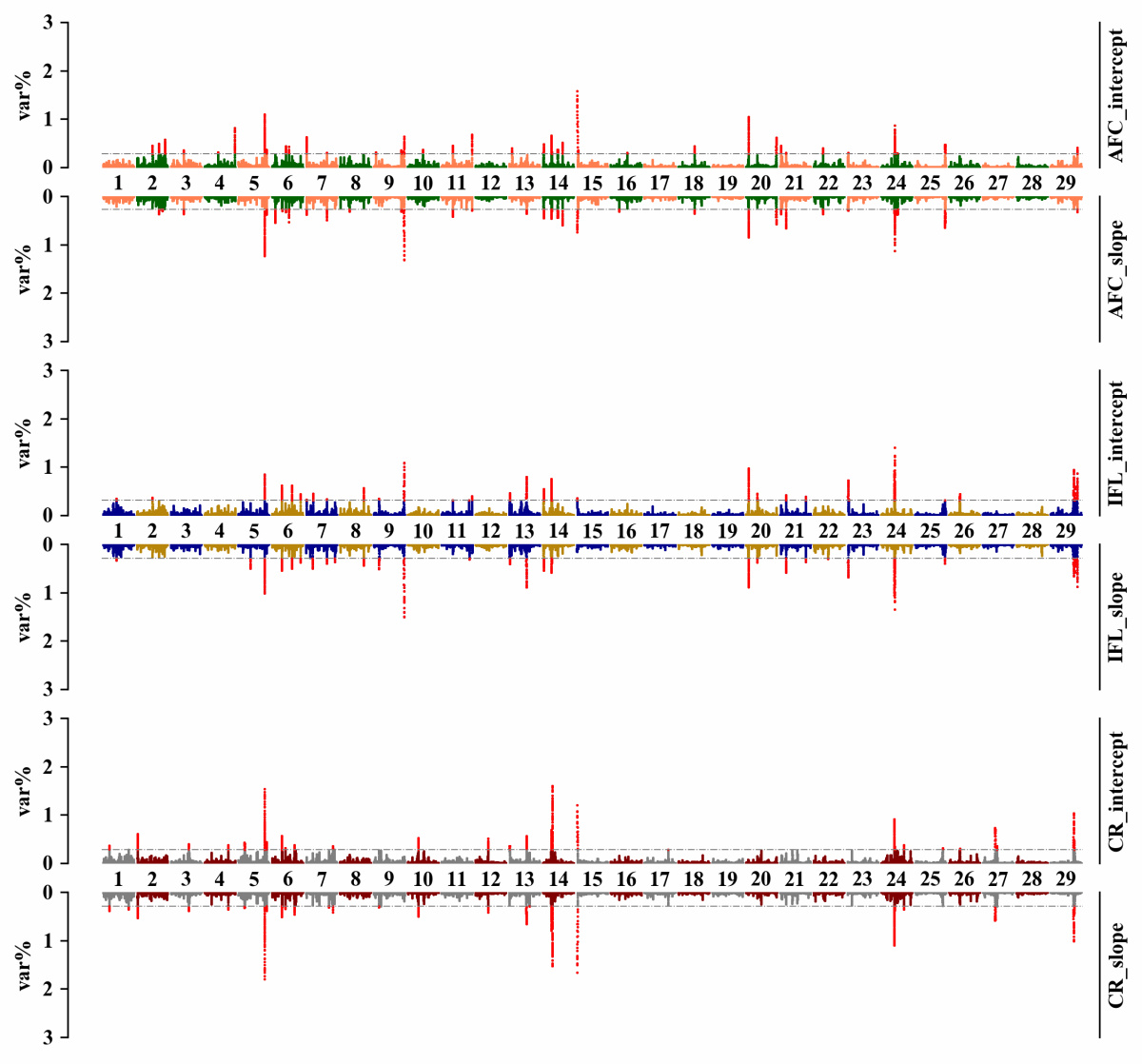

Supplement: Supplementary file 8 — Additional file 8: Figure S5. Percentages of the intercept and slope genetic variances explained by a sliding window of 20 SNPs for three traits, which were estimated under scenario two of mTHI-EG. [file 12864_2021_7496_MOESM8_ESM.jpg]

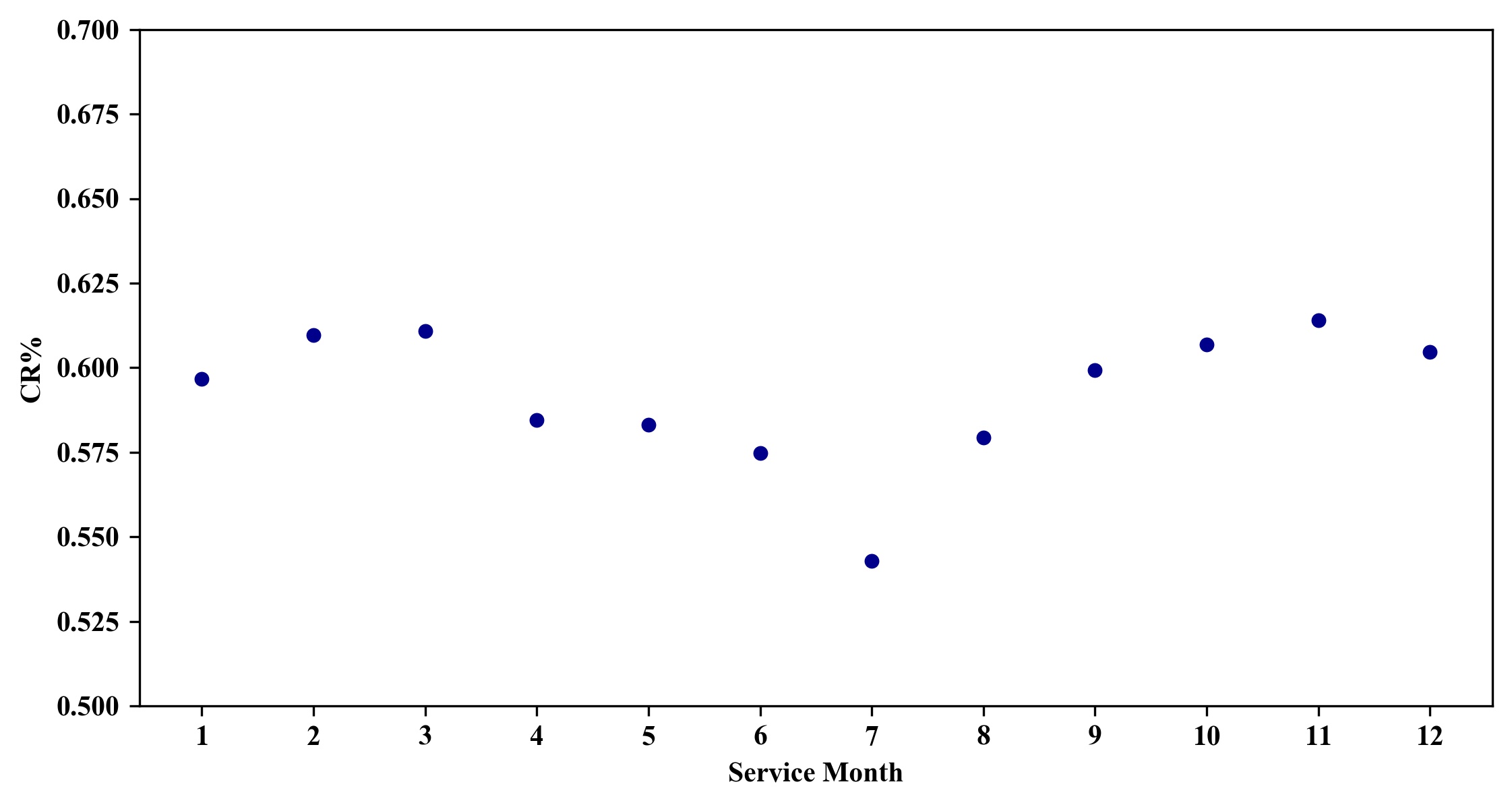

Supplement: Supplementary file 11 — Additional file 11: Figure S6. Average conception rate of Holstein population in different months. [file 12864_2021_7496_MOESM11_ESM.jpg]
